# Left Ventricular Geometry and Replacement Fibrosis Detected by cMRI Are Associated with Major Adverse Cardiovascular Events in Nonischemic Dilated Cardiomyopathy

**DOI:** 10.3390/jcm9061997

**Published:** 2020-06-25

**Authors:** Bianca Olivia Cojan-Minzat, Alexandru Zlibut, Ioana Danuta Muresan, Carmen Cionca, Dalma Horvat, Eva Kiss, Radu Revnic, Mira Florea, Razvan Ciortea, Lucia Agoston-Coldea

**Affiliations:** 1 Department of Internal Medicine, Iuliu Hatieganu University of Medicine and Pharmacy, 400006 Cluj-Napoca, Romania; cojanminzat.bianca@yahoo.com (B.O.C.-M.); alex.zlibut@yahoo.com (A.Z.); ioanamuresandanuta@yahoo.com (I.D.M.); hdalma92@yahoo.com (D.H.); kiss.eva24@yahoo.com (E.K.); r_ciortea@yahoo.com (R.C.); 2 Department of Family Medicine, Iuliu Hatieganu University of Medicine and Pharmacy, 400001 Cluj-Napoca, Romania; radu_revnic@yahoo.com (R.R.); mira_florea@yahoo.com (M.F.); 3 Department of Radiology, Affidea Hiperdia Diagnostic Imaging Center, 400015 Cluj-Napoca, Romania; carmen.cionca@yahoo.com; 4 Department of Obstetrics and Gynecology, Emergency County Hospital, 400124 Cluj-Napoca, Romania; 5 2nd Department of Internal Medicine, Emergency County Hospital, 400006 Cluj-Napoca, Romania

**Keywords:** nonischemic dilated cardiomyopathy, cardiac magnetic resonance imaging, late gadolinium enhancement, long axis strain, left ventricle sphericity index, major adverse cardiovascular events

## Abstract

To investigate the relationship between left ventricular (LV) long-axis strain (LAS) and LV sphericity index (LVSI) and outcomes in patients with nonischemic dilated cardiomyopathy (NIDCM) and myocardial replacement fibrosis confirmed by late gadolinium enhancement (LGE) using cardiac magnetic resonance imaging (cMRI), we conducted a prospective study on 178 patients (48 ± 14.4 years; 25.2% women) with first NIDCM diagnosis. The evaluation protocol included ECG monitoring, echocardiography and cMRI. LAS and LVSI were cMRI-determined. Major adverse cardiovascular events (MACEs) were defined as a composite outcome including heart failure (HF), ventricular arrhythmias (VAs) and sudden cardiac death (SCD). After a median follow-up of 17 months, patients with LGE+ had increased risk of MACEs. Kaplan-Meier curves showed significantly higher rate of MACEs in patients with LGE+ (*p* < 0.001), increased LVSI (*p* < 0.01) and decreased LAS (*p* < 0.001). In Cox analysis, LAS (HR = 1.32, 95%CI (1.54–9.14), *p* = 0.001), LVSI [HR = 1.17, 95%CI (1.45–7.19), *p* < 0.01] and LGE+ (HR = 1.77, 95%CI (2.79–12.51), *p* < 0.0001) were independent predictors for MACEs. In a 4-point risk scoring system based on LV ejection fraction (LVEF) < 30%, LGE+, LAS > −7.8% and LVSI > 0.48%, patients with 3 and 4 points had a significantly higher risk for MACEs. LAS and LVSI are independent predictors of MACEs and provide incremental value beyond LVEF and LGE+ in patients with NIDCM and myocardial fibrosis.

## 1. Introduction

Nonischemic dilated cardiomyopathy (NIDCM) is the most common primary myocardial disease, being characterized by left ventricular (LV) enlargement and global systolic LV function impairment in the absence of ischemic heart disease (IHD), hypertension or valve disease [1]. Due to its significant increased mortality [2] and sudden cardiac death (SCD) risk [3], NIDCM represents an important global healthcare burden. Nowadays, the development of more effective methods of assessing NIDCM severity and the risk of major adverse cardiovascular events (MACEs) remains a topic of great interest for current research.

While current guidelines recommend echocardiography as the first line of investigation in patients with NIDCM [4,5], it cannot evaluate the structural myocardial impairment [6]. Myocardial replacement fibrosis is part of the cardiac remodelling process, being responsible for heart failure (HF), ventricular arrhythmia (VA) and SCD. It is encountered in one-third of NIDCM patients, being detected using cardiac magnetic resonance imaging (cMRI) with late gadolinium enhancement (LGE) [7,8,9,10]. T1 mapping imaging is a state-of-the-art cMRI technique that is able to characterize extracellular volume fraction and it has been validated by comparative studies with the histopathological examination in NIDCM [11,12]. LV long-axis strain (LAS) determined by cMRI is an efficient and reliable method for quantifying global LV longitudinal function and it has an important prognostic value in patients with NIDCM [13,14]. Last but not least, cMRI-determined LV sphericity index (LVSI) is a parameter that predicts MACEs in NIDCM and it can be used in the assessment of LGE presence and LGE mass [15,16].

The aim of this study was to investigate the relationship between cardiac remodelling process and MACEs, and if it increases outcome prediction beyond LGE, in patients with NIDCM.

## 2. Methods

### 2.1. Study Population

**Figure 1:**
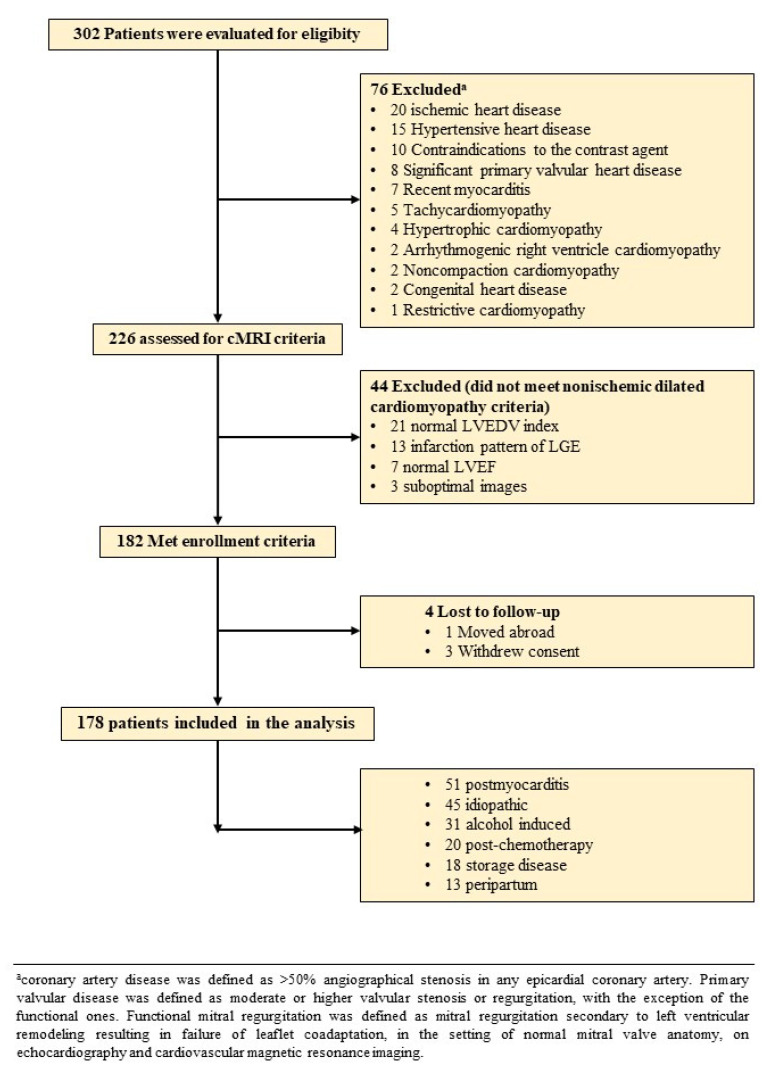
Flow chart detailing the identification of the study cohort. Abbreviations: cMRI, cardiac magnetic resonance imaging; LGE, late gadolinium enhancement; LVEDV, left ventricular end diastolic volume; LVEF, left ventricular ejection fraction.

We conducted a prospective study on 302 consecutive patients with first NIDCM diagnosis, which were examined in the 2th Department of Internal Medicine of the Iuliu Hatieganu University of Medicine and Pharmacy from October 2017 to November 2019. The inclusion criteria were [1]: (1) impaired global LV function with a LV ejection fraction (LVEF) ≤45%; (2) LV chamber dilation with an indexed LV end-diastolic volume (LVEDV) ≥97 mL/m^2^; both cMRI-determined. The exclusion criteria are represented by (1) IHD, other cardiomyophaties, significant valvular and congenital heart disease (CHD, CVD); (2) contraindications to cMRI (incompatible metallic devices, significant chronic renal disease with estimated glomerular filtration rate <30 mL/min/1.73 m^2^, or claustrophobia); (3) refusal to participate in the study (Figure 1). IHD was excluded by coronarography in 72 patients (41%), stress imaging studies in 64 patients (36%) and the remaining 42 patients (23%) had no history of angina, 1 or 0 risk factors for IHD and stress ECG test and computed tomography coronary angiography with Agatston calcium scoring were also negative.

We recorded demographic data including age, gender, height, weight, medical history, cardiovascular symptoms (dyspnoea, syncope, palpitations), and current medication; biomarkers and 12-lead ECG. 24-h Holter monitoring, transthoracic echocardiography and cMRI were performed. The current research was approved by the Ethics Committee of the Iuliu Hatieganu University of Medicine and Pharmacy, Cluj-Napoca—decision number 280/26.07.2018. The study was conducted in accordance with the principles of the Declaration of Helsinki. All patients were informed about the investigation protocol and signed a written consent form.

### 2.2. cMRI

All cMRI images were ECG-gated and were acquired during apnoea with a 1.5 T magnetic resonance (MR) scanner (Magnetom Symphony, Siemens Medical Solutions, Erlanger, Germany). A standard scanning protocol that was in accordance with current international guidelines was used [17]. The acquisition of fast imaging employing steady-state free precession (SSFP) sequences was performed to detect ventricular function and mass in the conventional cardiac short-axis and long-axis planes (including two-chamber, three-chamber, and four-chamber), to enclose both ventricles from base to apex. SSFP sequence parameters were as follows: repetition time (TR) 3.6 ms; echo time (TE) 1.8 ms; flip angle 60°; slice thickness 6 mm; field of view 360 mm; image matrix of 192 × 192 pixels; voxel size 1.9 × 1.9 × 6 mm; 25–40 ms temporal resolution reconstructed to 25 cardiac phases. LGE imaging was performed to detect focal myocardial scars acquired 10 min after intravenous administration of 0.2 mmol/kg gadoxetic acid (Clariscan, GH Healthcare AS, Oslo, Norway) in long- and short axis-views, using a segmented inversion-recovery gradient-echo sequence. LGE imaging sequence parameters were presented by: TR 4.8 ms, TE 1.3 ms, and inversion time 200 to 300 ms. Inversion time was adjusted to optimize nulling of apparently normal myocardium. Brachial blood pressure was monitored during cMRI-SSFP acquisitions.

**Figure 2:**
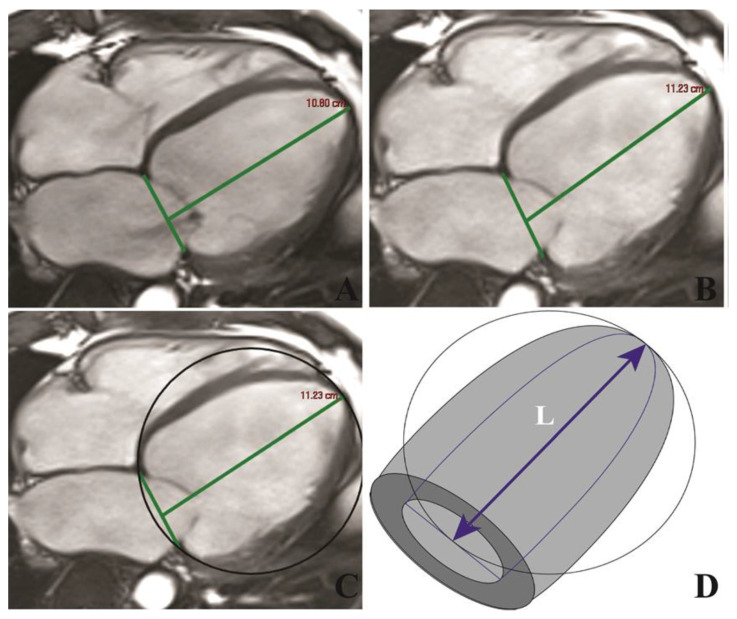
Representative image illustrating the technique for assessment of LAS (**A**,**B**) and LVSI (**C**,**D**) in a patient with severe NIDCM in end-diastole and end-systole, respectively. Abbreviations: LAS, left ventricular long axis strain; LVSI, left ventricular sphericity index; NIDCM, non-ischemic dilated cardiomyopathy.

Image analysis: All images were evaluated by two experienced observers, blinded to all clinical data. LVEDV and LV end-systolic volume (LVESV), LVEF and end-diastolic LV mass (LVM) were measured on short-axis cine-SSFP images. Epicardial and endocardial borders were traced semi-automatically at end-diastole and end-systole using specialized software (Syngo.Via VB20A_HF04, Argus, Siemens Medical Solutions). The maximum left atrium (LA) and right atrium (RA) volumes were measured in all patients from the four-chamber view. All volumes were indexed to body surface area. Tricuspid annular plane systolic excursion (TAPSE) was measured from the mid-four-chamber cardiac view to assess right ventricular (RV) longitudinal motion. LV longitudinal function was assessed by LAS, defined as the difference in mitral annular displacement at end-systole vs. end-diastole, and expressed as a percentage [13]. LVSI was calculated by dividing LVEDV to the volume of a sphere whose LV length (L) is measured at end-diastole: LVSI = LVEDV/(π/6 × (L)^3^) [15] (Figure 2).

The presence and distribution of LGE in the LV were assessed from short-axis images, using the 17-segments model, as recommended by the American Heart Association [18], and were quantified using a signal intensity threshold of >5SD above a remote reference for normal myocardium. Due to the fact that the LGE quantification with the threshold of 5SD demonstrated the best agreement with visual assessment and best reproducibility among different technique thresholds, we used a threshold of 5SD above the signal intensity of normal myocardium [19,20]. LGE’s distribution was characterized as mid-wall, subepicardial, focal or diffuse. The assessment of LGE mass in the LV was automatically quantified from short-axis LGE images using cardiac dedicated software (cvi42, Circle Cardiovascular Imaging Inc., Calgary, CA). The extent of LGE was expressed by gram (g) and also as percentage of LVM. According to the cMRI, the studied population was divided into two groups, namely: patients without LGE (LGE−) and patients with LGE (LGE+).

### 2.3. Follow-Up of Clinical Outcomes

The clinical follow-up was obtained by completing a questionnaire either on hospital visits, telephone house-calls, or both, aiming at delineating the occurrence of the clinical outcomes, which corresponded to the first event occurring in each patient among the following MACEs: death or aborted death from cardiac cause, sustained ventricular tachyarrhythmia (beats with ventricular origin that lasts >30 s and has a rate greater than >100 beats/min), and HF requiring hospitalization defined accordingly to current international guidelines [4,5]. Hospitalisation due to non-cardiac causes was not counted as event. Survival analysis was performed for the clinical outcomes. The median follow-up was 17 months and maximum follow-up reached 29 months.

### 2.4. Statistical Analysis

All data were tested for normality using the Kolmogorov-Smirnov test. Data were presented as median, mean ± standard deviation (SD) or percentage. Baseline characteristics among those with and without LGE patients with clinical outcomes were compared by Chi-square χ^2^ test or Fischer exact test as appropriate (categorical data) and Wilcoxon signed rank test (continuous data). The hazard ratio (HR) for the prediction of events was calculated using a Cox regression model. For each outcome, we considered all of the significant variables in the univariate analysis and sought the best overall multivariate models for the composite end-point, by stepwise-forward selection, with a probability to enter set at *p* < 0.05 and to remove the effect of regression at *p* < 0.05. Event-free survival was generated by the Kaplan–Meier method and statistical significance was determined by the log-rank test. Multivariate analysis was performed by constructing a multiple logistic regression model, including the HR (95%CI) calculation. Cohen’s Kappa inter- and intra-observer coefficient calculation was performed. Retrospective test power calculation and prospective sample size were estimated, with type I and type II variation according to sample size. The statistical analysis was performed using the MedCalc (Version 19.1.7, MedCalc Software, Ostend, Belgium).

## 3. Results

### 3.1. Baseline Characteristics

A total of 178 patients (48 ± 14.4 years old, 74.7% male) met the enrolment criteria (Figure 1). They were divided in two groups according to LGE+ and LGE− (*n* = 64, 36% vs. *n* = 114, 64%). The baseline characteristics are presented in Table 1. 

At admission, 54 (30.3%) presented with dyspnoea, 28 (15.7%) with palpitations and 18 (10.1%) had history of syncope. LGE+ patients had significantly increased LV end-diastolic filling pressures (E/E’ ratio; *p* < 0.001) and many more of these had LVEF < 30% (*n* = 46, 71.8%). The LGE+ group presented with increased LVM index ≥ 92 g/m^2^ (*p =* 0.01), LVEDV ≥ 145.5 mL/m^2^ (*p* < 0.001), LVSI ≥ 0.43 (*p* < 0.001) and reduced LAS < −7.8% (*p* < 0.001).

### 3.2. Reproducibility of cMRI Measurements

cMRI measurements were repeatedly performed on the same set of images, acquired from all patients in the study group. Regarding LVEF, LAS, LVSI and the assessment of LGE+, the intra- and inter-observer reproducibility were excellent. The inter-observer kappa coefficients of agreement were 0.91 for LVEF, 0.97 for LAS, 0.93 for LVSI and 0.88 for LGE+, while the intra-observer kappa coefficients of agreement were 0.98 for LVEF, 0.98 for LAS, 0.92 for LVSI and 0.90 for LGE+ (Table 2).

### 3.3. Survival Analysis

**Figure 3:**
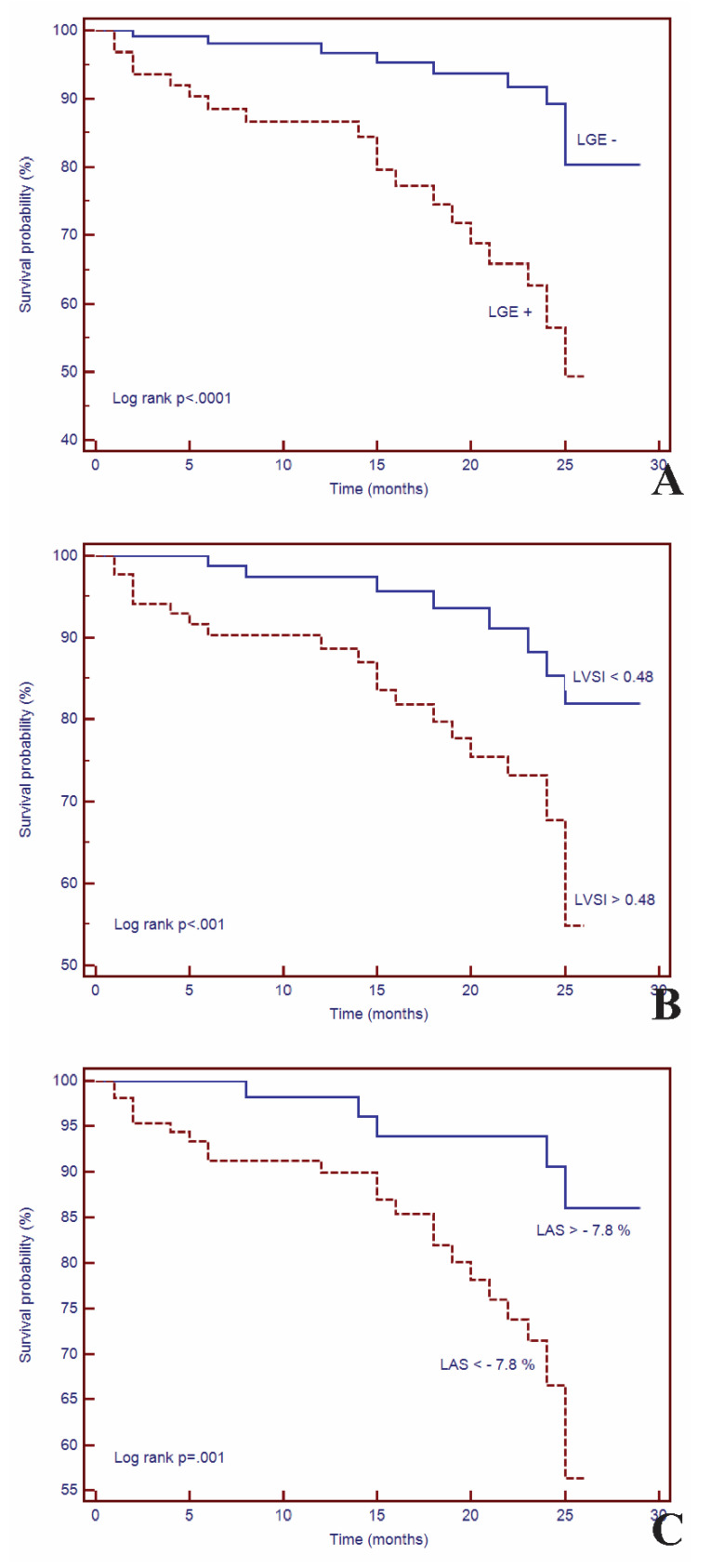
Kaplan–Meier curves for event-free survival for (**A**) LGE+, (**B**) LAS, (**C**) LVSI. Abbreviations: LAS, long axis strain; LGE, left ventricular late gadolinium enhancement; LVSI, left ventricular sphericity index.

Initial cMRI evaluation was performed. During a median follow-up of 17 months (IQR 1 to 29 months), 31 patients (17.4%) experienced MACEs: VA (*n* = 14), HF requiring hospitalization (*n* = 11), and SCD (*n* = 6). The patients with VA were majority males, had increased LVESV (mean 107,533 mL, *p* < 0.001), decreased LVEF (28,429%, *p* < 0.0001), increased LGE mass (24,5 g, *p* < 0.0001) and LVSI (0.46, *p* < 0.001). Of them, ten received ICD therapy and 4 were ablated due to implantable cardioverter defibrillator therapy refusal, the last had a mean LVEF around 30%, two of them had NIDCM post-myocarditis and two were idiopathic NIDCM. The incidence of MACEs was significantly higher in the LGE+ group vs. the LGE− group (*n* = 21, 67.7% vs. *n* = 10, 33.4%). The Kaplan-Meier curves for event-free survival showed a significantly higher rate of MACEs in patients with LGE+ (HR = 4.02; 95%CI (1.91–8.45), *p* < 0.001), high LVSI (HR = 3.23; 95%CI (1.59–6.53), *p* < 0.01) and decreased LAS (HR = 3.94; 95%CI (1.93–8.03), *p* < 0.001) (Figure 3).

### 3.4. Univariate and Multivariate Cox Analysis

Among the evaluated parameters, in univariate analysis and multivariate Cox regression analysis, only four remained independent predictors for MACEs, namely LGE+ (HR = 1.77, 95%CI (2.79 to 12.51), *p* < 0.0001), reduced LAS (HR = 1.32, 95%CI (1.54 to 9.14), *p* < 0.001) and increased LVSI (HR = 1.17, 95%CI (1.45 to 7.19), *p* < 0.001) and LGE mass (HR = 1.43, 95%CI (1.01–6.12), *p* < 0.001) (Table 3).

### 3.5. Incremental Predictive Value of cMRI-Based LV Geometry and Strain for Outcomes

**Figure 4:**
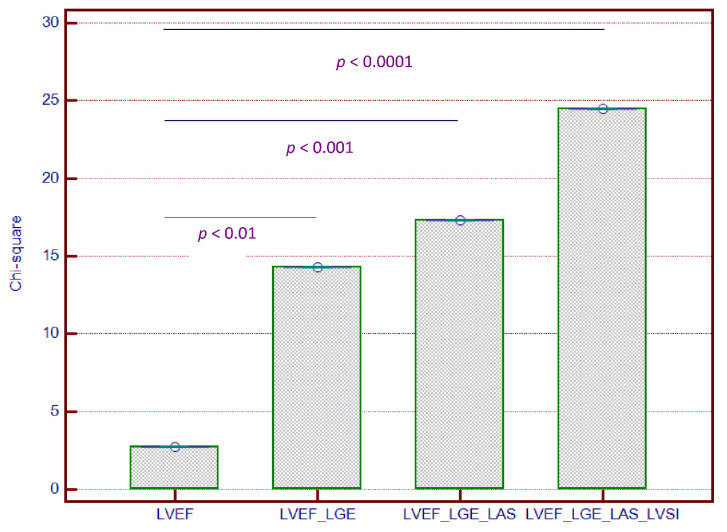
Incremental predictive value of LVSI and LAS added to LVEF and to LGE for outcome in patients with NIDCM. Abbreviations: LAS, left ventricular long axis strain; LGE, left ventricular late gadolinium enhancement; LVSI, left ventricular sphericity index; NIDCM, nonischemic dilated cardiomyopathy.

Sequential Cox proportional-hazards models yielded significantly increased predictive power the combined outcome of MACEs when both LVSI and LAS were used in addition to LVEF and LGE+ (Chi-square = 24.52, *p* < 0.0001) (Figure 4). However, LAS did not provide incremental predictive power when used alone, in addition to LVEF and LGE+.

### 3.6. Risk Stratification Scoring System

**Figure 5:**
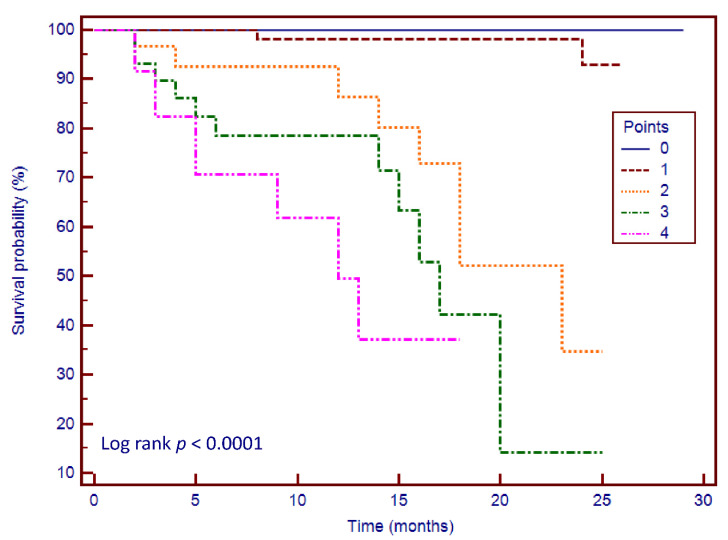
Kaplan-Meier curves for the risk stratification score. The scoring system ranges from 0 to 4 points: 1 point for each of the following LVEF < 30%, LGE+, LAS > −7.8% and LVSI > 0.48. Abbreviations: LAS, left ventricular long axis strain; LGE, left ventricular late gadolinium enhancement; LVSI, left ventricular sphericity index.

The embedment of LVSI and LAS to LVEF and LGE allowed us to create a risk stratification score, using the following criteria: LVEF < 30%, LGE+, LVSI > 0.48 and LAS < −7.8%. These cut-off values were best correlated with outcome in our studied group. We created a scoring system and Kaplan–Meier curves based on the four parameters (Chi-square = 56.53, *p* < 0.0001) (Figure 5). We observed that patients with 3–4 points had significantly higher rates of MACEs during the follow-up period than others.

## 4. Discussion

This prospective study is the first to evaluate the association between cMRI-based LV geometry and strain and outcome, in a significant, well-diagnosed NIDCM cohort. LAS and LVSI were independent predictors of MACEs in patients with NIDCM and myocardial replacement fibrosis. These findings were independent of LVEF and other established prognostic factors in a multivariable analysis. We also demonstrated that the addition of both LAS and LVSI to LVEF and LGE was superior for the prediction of MACEs over those based only on LVEF and LGE. The incidence of MACEs was higher in those with myocardial replacement fibrosis and altered LV geometry and strain, therefore representing a group who may require more aggressive therapy and rigorous follow-up.

NIDCM is typically associated with LV mid-wall replacement fibrosis, which worsens its prognosis [21,22]. Furthermore, Lehrke et al. have shown that NIDCM can be confirmed by cMRI-determined LV midwall fibrosis [23]. In our study, the incidence of LGE in NIDCM was similar with other studies [8,24] and, in addition, we demonstrated that LGE was an independent predictor of MACEs. Similarly, in patients with NIDCM, Gulati et al. showed that the presence of mid-wall LGE was an independent predictor for outcome and improved risk stratification beyond LVEF [9]. Furthermore, several studies have confirmed that the presence LGE is independently associated with all-cause mortality, SCD and aborted SCD [25,26].

In our study, we confirmed the role of LVSI in predicting MACEs with a cut-off value of >0.43 (*p* < 0.001), similar with other published data. In patients with NIDCM, LVSI was initially evaluated in 2- and 3-dimensional echocardiography-based studies, which demonstrated that it was an independent predictor of MACEs, having a significant long-term prognostic impact [15,27]. Furthermore, in cMRI-based studies, it has been confirmed that LVSI is inversely correlated with LVEF in patients with NIDCM [28,29], while in a multi-ethnic study conducted on healthy subjects, lowest LVSI was an independent predictor for CHD, CVD and HF at 10-year follow-up and the highest LVSI was correlated with increased incidence of HF and atrial fibrillation [30]. Moreover, in a small study, LVSI was an independent predictor for correct ICD therapy [31]. Thereby, LVSI could become an important prediction parameter in this category of patients.

Furthermore, we demonstrated that LAS, a myocardial strain parameter, was an independent predictor for outcome in patients with NIDCM. cMRI-based myocardial strain has proved its utility in early diagnosing and predicting various cardiac diseases. In patients with myocardial infarction, Gjesdal et al. showed that LAS was progressively reduced in larger mitral insufficiency and was associated with the infarction mass [32], while Schuster et al. identified that the assessment of LAS provided incremental prognostic value for cardiovascular risk [33]. In a multi-ethnic study, LAS was also associated with LVEF and MACEs [34]. In a study conducted on patients with aortic stenosis, our research team identified that LAS was an independent predictor of outcome and provided incremental value beyond LVEF and LGE [35]. Lastly, in patients with NIDCM, a single study identified that LAS was an independent predictor for SCD, aborted SCD, heart transplantation and HF hospitalization [36].

The role of LVEF and LGE as independent predictors of MACEs in patients with NIDCM has been confirmed by recently published data. To our knowledge, only two studies have approached our goals, namely Kano et al. identified that the addition of LVSI to LGE significantly increased prognosis of MACEs [37], while Riffel et al. demonstrated that the addition of LAS to LGE provides incremental value for outcome prediction in patients with NIDCM [36]. Our investigation is the first to demonstrate that the combined addition of both LVSI and LAS to LVEF and LGE significantly increased the predictive power of outcome, thus conferring an incremental predictive value.

Based on these four parameters, we were able to create a risk stratification scoring system. Hitherto, a single study created a similar scoring system based on LAS, LVEF and LGE+ which provided significant predictive value [36]. We demonstrated that the addition of one point for each of these parameters (LVEF < 30%, LGE+, LAS > −7.8% and LVSI > 0.48) is highly correlated with MACEs. In patients without these risk features (score = 0), no MACEs were observed during follow-up. Thereby, we propose a combined risk score consisting of LVEF, LGE, LVSI and LAS in order to improved risk stratification.

Study limitations: Firstly, we conducted a single centre study. Secondly, we were unable to acquire T1 mapping sequences, and therefore extracellular volume and diffuse myocardial fibrosis could not be quantified. Additionally, the follow-up was, relatively speaking, not very long.

## 5. Conclusions

cMRI parameters of geometry and longitudinal strain, namely LVSI and LAS, are independently associated with increased risk of MACEs in NIDCM patients with myocardial replacement fibrosis confirmed by cMRI-LGE. For the first time, we demonstrate that the combined usage of LVSI and LAS provide incremental value beyond the assessment of LVEF and LGE in outcome prediction. These findings have potential therapeutic implications regarding the management of patients with NIDCM.

## Figures and Tables

**Table 1:** Baseline characteristics of patients in study.

	All Patients *n* = 178	LGE− *n* = 114	LGE+ *n* = 64	*p*-Value
**Clinical characteristics**				
- Age, mean (SD), years	48 (14.4)	47 (15.0)	45 (13.4)	NS
- Male gender, *n* (%)	133 (74.7)	83 (72.8)	50 (78.1)	NS
- Body-mass index, kg/m^2^	27.4 (4.8)	27.3 (4.4)	27.5 (5.7)	NS
- Heart rate, mean (SD), bpm	73 (16.0)	71 (14.3)	76 (18.3)	<0.05
- Systolic blood pressure, mean (SD), mmHg	133 (18.9)	134 (18.4)	130 (19.5)	NS
- Hypertension, *n* (%)	98 (55.1)	69 (60.5)	29 (45.5)	<0.05
- Diabetes mellitus, *n* (%)	58 (32.5)	41 (35.9)	17 (26.5)	NS
- Dyslipidemia, *n* (%)	104 (58.4)	67 (58.8)	37 (57.8)	NS
- Smoking, *n* (%)	58 (32.5)	39 (34.2)	19 (29.6)	NS
- NYHA functional class I/II/III, *n*	29/59/27	19/39/19	10/20/8	<0.05
**Electrocardiogram**				
- Atrial fibrillation, *n* (%)	26 (14.6)	18 (15.7)	8 (12.5)	NS
- Left bundle branch block, *n* (%)	15 (8.4)	10 (8.7)	5 (7.8)	NS
- Right bundle branch block, *n* (%)	17 (9.5)	12 (10.5)	5 (7.8)	NS
- Significant Q waves, *n* (%)	21 (11.8)	14 (12.3)	7 (10.9)	NS
**Medications**				
- Beta-blockers, *n* (%)	142 (79.7)	91 (79.8)	51 (79.6)	NS
- ACEIs or ARBs, *n* (%)	130 (73.0)	84 (73.6)	46 (71.8)	NS
- Calcium channel blockers, *n* (%)	28 (15.7)	18 (15.7)	10 (15.6)	NS
- Statins, *n* (%)	105 (58.9)	67 (58.7)	38 (59.3)	NS
- Antiplatelet therapy, *n* (%)	68 (38.2)	44 (38.5)	25 (37.5)	NS
- Diuretics, *n* (%)	111 (62.3)	70 (61.4)	41 (64.0)	NS
- Digitalis, *n* (%)	13 (7.3)	8 (7.0)	5 (7.8)	NS
**Biomarker levels**				
- NT-proBNP, median (IQR), pg/mL	2639.5 (378–11,960)	2600 (378–9893)	2679 (570–11,960)	NS
- eGFR, mean (SD), ml/min/1.73 m^2^	87.1 (21.2)	87.7 (20.4)	86.1 (22.6)	NS
**Echocardiography**				
- E/E’ ratio, mean (SD)	9.27 (2.5)	8.2 (2.4)	12.3 (2.6)	<0.001
- DT, mean (SD), ms	217 (56.7)	215 (53.4)	221 (63.1)	NS
- sPAP, mean (SD), mmHg	30.8 (12.0)	30.1 (12.5)	32.2 (11.0)	NS
**Cardiovascular magnetic resonance**				
- LVEDV index, mean (SD), mL/m^2^	132.3 (34.5)	124.8 (30.2)	145.5 (37.8)	<0.001
- LVESV index, mean (SD), mL/m^2^	87.5 (34.4)	78.6 (29.6)	103.5 (36.8)	<0.001
- LVM index, mean (SD), g/m^2^	86.7 (20.6)	83.7 (19.6)	92.0 (21.6)	<0.01
- LVEF, mean (SD), %	35.0 (9.3)	37.8 (7.7)	29.9 (9.7)	<0.001
- LAV index, mean (SD), mL/m^2^	55.8 (21.3)	53.1 (20.4)	60.6 (22.2)	<0.05
- LAS, mean (SD), %	−9.6 (5.3)	−10.7 (5.4)	−7.8 (4.6)	<0.001
- LVSI, mean (SD)	0.40 (0.12)	0.38 (0.11)	0.43 (0.13)	<0.001
- TAPSE, mean (SD), mm	18.6 (5.2)	19.5 (5.3)	16.9 (4.7)	0.001
- RVEDV index, mean (SD), mL/m^2^	53.4 (21.2)	52.7 (19.2)	54.7 (24.4)	NS
- RVESV index, mean (SD), mL/m^2^	29.0 (15.5)	27.2 (11.9)	32.2 (20.0)	<0.01
- RVEF, mean (SD), %	46.8 (9.55)	49.0 (8.7)	42.8 (9.7)	<0.01
- LV-LGE mass median (IQR), g			30.5 (1–88)	
- LV-LGE mass/LVM, median (IQR), %			17.2 (0.6–54)	

Abbreviations: ACEI, angiotensin converting enzyme inhibitor; ARB, angiotensin receptor blocker; DT, early diastolic filling deceleration time; E, peak mitral flow velocity; E’, early diastolic peak myocardial velocity; eGFR, estimated glomerular filtration rate; IQR, interquartile range; LAS, left ventricular longitudinal-axis strain; LAV, left atrial volume; LGE, left ventricular late gadolinium enhancement; LVEDV, left ventricular end-diastolic volume; LVEF, left ventricular ejection fraction; LVESV, left ventricular end-systolic volume; LVM, left ventricular mass; LVSI, left ventricular sphericity index; *n*, number of patients; NT-proBNP, N-terminal pro-Brain Natriuretic Peptide; NYHA, New York Heart Association; RVEDV, right ventricular end-diastolic volume; RVEF, right ventricular ejection fraction; RVESV, right ventricular end-systolic volume; SD, standard deviation; sPAP, systolic pulmonary artery pressure; TAPSE, tricuspid annular plane systolic excursion. Data are reported as mean (standard deviation) or median (IQR) or *n* (%).

**Table 2:** Reproducibility inter and intra-reader agreement of cMRI measurements.

Parameters	Coefficient Kappa	95% Confidence Interval	Standard Error
Inter-observer			
LVEF	0.91	0.872 to 0.941	0.026
LAS	0.97	0.909 to 0.989	0.012
LGE	0.88	0.771 to 0.939	0.066
LVSI	0.93	0.856 to 0.952	0.029
Intra-observer			
LVEF	0.98	0.977 to 0.992	0.009
LAS	0.98	0.967 to 0.991	0.004
LGE	0.90	0.835 to 0.948	0.023
LVSI	0.92	0.871 to 0.928	0.032

Abbreviations: LAS, left ventricular longitudinal-axis strain; LGE, left ventricular late gadolinium enhancement; LVEF, left ventricular ejection fraction; LVSI, left ventricular sphericity index.

**Table 3:** Univariable and multivariable Cox analysis testing between studied parameters and MACEs.

	No Events *n* = 147	Events *n* = 31	Univariable Analysis		Multivariable Analysis	
			Unadjusted HR (95% CI)	*p* Value	Adjusted HR (95% CI)	*p* Value
Age, years	48 (13.8)	48 (17.5)	1.00 (0.98–1.03)	NS		
Male gender, n, %	111 (75.5)	22 (37.9)	1.14 (0.53–2.48)	NS		
Body-mass index, kg/m^2^	27.7 (4.8)	25.8 (4.5)	0.94 (0.87–1.01)	NS		
Systolic blood pressure	134 (19.2)	130 (17.4)	0.99 (0.97–1.01)	NS		
NT-proBNP, pg/mL	2564 (378–11960)	2834 (834–9892)	1.00 (0.99–1.01)	NS		
eGFR, ml/min/1.73 m^2^	86.3 (20.1)	91.0 (25.9)	1.01 (0.97–1.03)	NS		
LVEDV index, mL/m^2^	131.4 (35.6)	136.6 (32.7)	1.01 (0.99–1.01)	NS		
LVESV index, mL/m^2^	86.4 (34.5)	93.1 (33.8)	1.05 (0.98–1.07)	NS		
LVM index, g/m^2^	87.0 (20.7)	85.2 (20.8)	0.99 (0.97–1.01)	NS		
LVEF, %	35.5 (9.2)	32.4 (9.4)	0.97 (0.93–1.01)	NS		
LAV index, mL/m^2^	54.7 (21.7)	61.3 (18.6)	1.01 (1.00–1.03)	NS		
LGE+	43 (29.2)	21 (67.7)	4.03 (1.90–8.52)	<0.0001	1.77 (2.79–12.51)	<0.0001
LGE mass, g	11.3 (10.6)	28.8 (19.3)	1.23 (1.90–4.52)	<0.0001	1.43 (1.01–6.12)	<0.001
LAS, %	−10.0 (5.6)	−7.8 (3.6)	1.19 (1.01–2.18)	<0.001	1.32 (1.54–9.14)	0.001
LVSI, %	0.38 (0.11)	0.48 (0.13)	2.13 (1.05–8.11)	<0.001	1.17 (1.14–7.19)	<0.01
E/E’ ratio	9.1 (2.3)	15.7 (4.8)	1.08 (0.95–1.22)	<0.05	1.02 (0.92–1.01)	NS
TAPSE, mm	18.8 (5.2)	17.4 (5.4)	0.77 (0.70–0.84)	NS		
RVEDV index, mL/m^2^	53.3 (19.7)	53.8 (27.6)	1.00 (0.98–1.02)	NS		
RVESV index, mL/m^2^	28.2 (13.5)	32.4 (13.9)	1.02 (1.00–1.04)	NS		
RVEF, %	47.5 (9.3)	43.4 (10.2)	0.84 (0.79–0.88)	NS		

Abbreviations: E, peak mitral flow velocity; E’, early diastolic peak myocardial velocity; eGFR, estimated glomerular filtration rate; IQR, interquartile range; LAS, left ventricular longitudinal-axis strain; LAV, left atrial volume; LGE, left ventricular late gadolinium enhancement; LVEDV, left ventricular end-diastolic volume; LVEF, left ventricular ejection fraction; LVESV, left ventricular end-systolic volume; LVM, left ventricular mass; LVSI, left ventricular spherical index; *n*, number of patients; NT-proBNP, N-terminal pro-Brain Natriuretic Peptide; RVEDV, right ventricular end-diastolic volume; RVEF, right ventricular ejection fraction; RVESV, right ventricular end-systolic volume; TAPSE, tricuspid annular plane systolic excursion.
